# Toxic Epidermal Necrolysis and Recurrent Implantation Failure: Unveiling a Rare Phenomenon During Pregnancy

**DOI:** 10.1002/ccr3.71226

**Published:** 2025-10-13

**Authors:** Hatav Ghasemi Tehrani, Faezeh Zakerinasab, Negar Khalili Geshnigani, Rahem Rahmati, Nastaran Zamani Dehkordi

**Affiliations:** ^1^ Department of Obstetrics, Gynecology, and Infertility School of Medicine, Isfahan University of Medical Sciences Isfahan Iran; ^2^ Mashhad University of Medical Sciences Mashhad Iran; ^3^ Students Research Committee Shahrekord University of Medical Sciences Shahrekord Iran

**Keywords:** hydroxychloroquine, in vitro fertilization, infertility, obstetrics and gynecology, TEN

## Abstract

Hydroxychloroquine (HCQ) is a common treatment for recurrent implantation failure (RIF), which may be associated with diverse side effects. We report a case of a 36‐year‐old RIF3 pregnant female through in vitro fertilization (IVF) presented to the emergency department with painful, itchy, macular, erythematous, blanchable, and exfoliative skin lesions distributed on the face, upper trunk, and upper limbs. She had been prescribed HCQ about 10 days before embryo transfer for her RIF history. With suspicion of Stevens–Johnson syndrome/Toxic epidermal necrolysis (TEN), HCQ was discontinued, and intravenous immunoglobulins (IVIg), triamcinolone, chlorpheniramine, and body lotion were administered. Considering more than 30% of the total body surface area involvement, within 3 days of treatment, and with favorable improvements, the diagnosis of TEN was confirmed. With the rising popularity of HCQ in immune‐related conditions, it is essential to highlight the significance of continuous monitoring for uncommon adverse reactions. The need for vigilance becomes particularly critical in patients requiring immediate intervention to prevent irreversible consequences.

**Summary**

- Hydroxychloroquine can cause severe adverse reactions, such as Stevens–Johnson syndrome and toxic epidermal necrolysis, even in patients with recurrent implantation failure who become pregnant while undergoing treatment.
- Continuous monitoring for uncommon side effects is crucial to ensure timely intervention and prevent serious complications.

**Abbreviations**
AGEP: acute generalized exanthematous pustulosis
HCQ: hydroxychloroquine
IVF: in vitro fertilization
IVIg: intravenous immunoglobulins
RIF: recurrent implantation failure
SJS: Stevens–Johnson syndrome
TEN: toxic epidermal necrolysis

## 1 Introduction

Recurrent implantation failure (RIF) refers to three or more in vitro fertilization (IVF) attempts using morphologically good embryos and a normal uterus, which do not result in successful implantation and clinical pregnancy [1]. According to the immunologic explanations of RIF, several immune‐based treatments have been suggested. Among them, hydroxychloroquine (HCQ) is commonly used as a treatment for RIF, which may be associated with various cutaneous side effects, including acute generalized exanthematous pustulosis (AGEP), Stevens‐Johnson syndrome (SJS), and toxic epidermal necrolysis (TEN) [2, 3]. This report is about a pregnant 36‐year‐old patient with RIF who developed cutaneous lesions.

## 2 Case History/Examination

**FIGURE 1:**
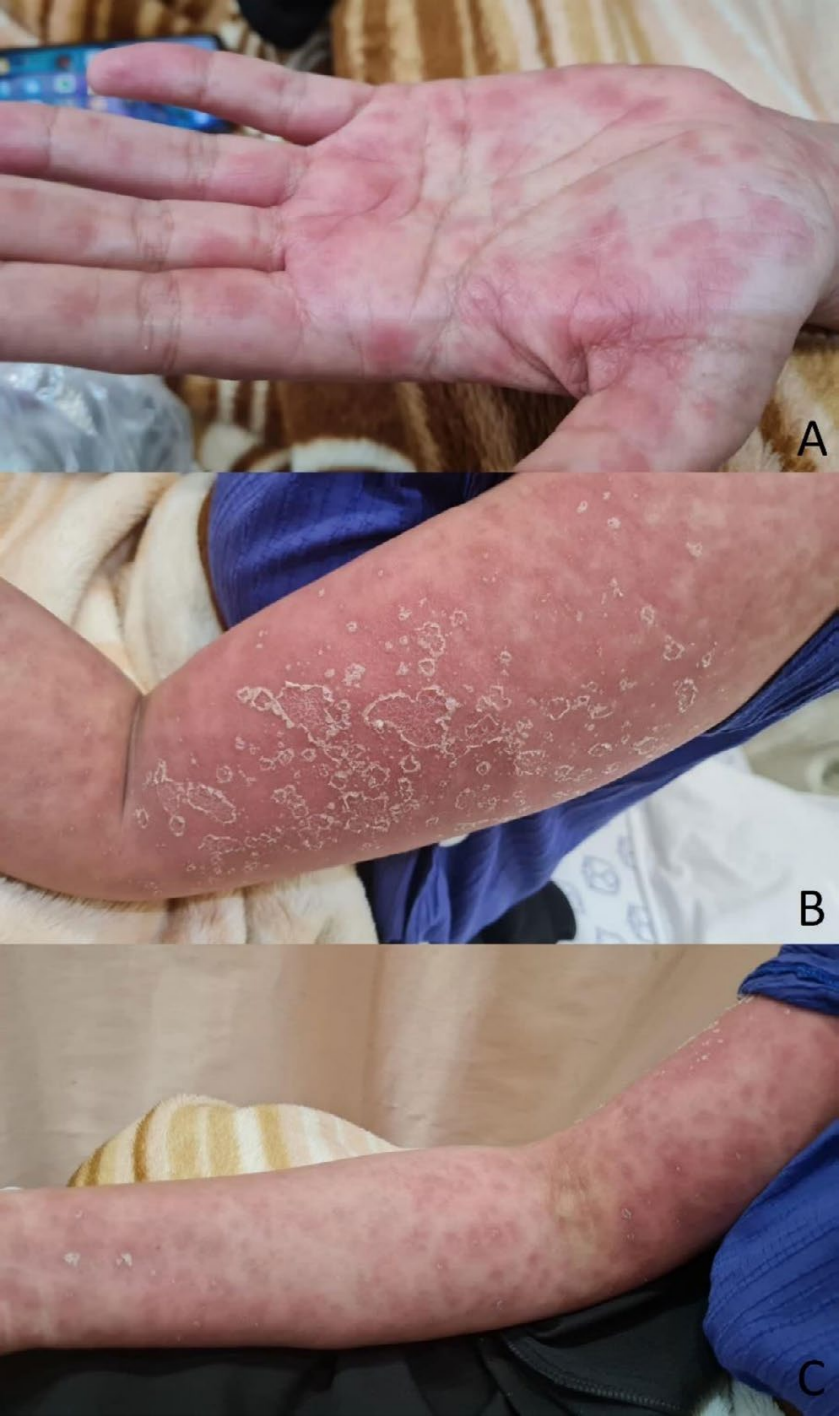
Skin lesions. (A) Hand, (B) Arm, (C) Arm and forearm.

A 36‐year‐old RIF3 pregnant woman came to the emergency department with complaints of aggravated painful, itchy, and desquamative skin lesions persisting for about a week. At that time, she was 5 weeks and 5 days pregnant with twins conceived via IVF. The lesions were painful, erythematous, macular, blanchable, and exfoliative. They initially appeared diffusely over the face, then the upper trunk, and extended to the upper limbs. More than 30% of the patient's total body surface area was affected by these lesions (Figure 1). Additionally, subcutaneous edema was observed, and a crust was present on her vermilion lips. There were no blisters, urticaria, ulcers, pustules, or angioedema, and the Nikolsky sign was positive. She did not recently experience fever, chills, nausea, vomiting, or other related symptoms.

As a consequence of her previous RIF encounter approximately 2 years ago, the patient sought consultation with an immunologist prior to her present embryo transfer. Approximately 10 days prior to the embryo transfer, HCQ (200 mg twice daily) was prescribed. Subsequently, to prime the patient for the embryo transfer procedure, a medication regimen consisting of daily aspirin 80 mg, estradiol 6 mg, and prednisolone 5 mg was initiated from the third day of her menstrual cycle until the attainment of a 7 mm endometrial thickness. Once the desired endometrial thickness was achieved and the transfer was completed, progesterone was introduced. The patient is not a smoker or tobacco user and has no history of alcohol use. Also, the patient reported no history of recent travel or exposure to ill contacts.

## 3 Methods (Differential Diagnosis, Investigations, and Treatment)

One week prior to her arrival at the emergency department, the patient presented at the clinic with newly observed painless macular lesions on her facial skin. During this visit, considering the potential reaction to progesterone, the lesions were initially postulated to be progesterone‐induced. Consequently, progesterone administration was halted, and human chorionic gonadotropin was prescribed as an alternative. However, the lesions continued progressing until the patient arrived at the emergency department.

During the physical examination, the patient was ill with an axillary temperature of 37°C, pulse rate of 95 beats/min, a respiratory rate of 30, and blood pressure of 100/75. Except for skin conditions and general aches and pains, no other pathologic signs had been detected. Based on the history and examination, laboratory tests were ordered due to suspicions of infections, SJS, and TEN. Meanwhile, transvaginal sonography was performed to evaluate the twins and examine the endometrium. The radiologist's report indicated that both fetuses were in satisfactory condition at 6 weeks, with no observed abnormalities.

## 4 Conclusions and Results (Outcome and Follow‐Up)

The patient's laboratory results revealed that the differential cell blood count, erythrocyte sedimentation rate, C‐reactive protein, liver function tests, blood urea nitrogen, creatinine, and urine analysis were within normal limits. Consequently, based on the patient's medical history, physical examination, and laboratory findings, consultation with an immunologist and dermatologist led to the decision to discontinue HCQ without replacement medication, pri and intravenous immunoglobulins (IVIg) administered for the patient. Additionally, topical cream of triamcinolone twice daily, chlorpheniramine daily, and body lotion were prescribed.

Following a favorable improvement over 3 days and more than 30% of total body surface area involvement, a TEN diagnosis was confirmed, and the patient was discharged with prednisolone, triamcinolone cream, loratadine, progesterone, chlorpheniramine, and body lotion. The patient attended follow‐up appointments 1 week and 2 weeks after discharge without serious problems. Due to maternal pain, the patient's delivery was a cesarean section at 36 weeks' gestation. The neonates, a boy and a girl, exhibited APGAR scores of 9 and 10, respectively. Both the mother and the newborns were discharged in a favorable general condition. No serious problems have been reported following a month of follow‐up visits.

## 5 Discussion

Infertility impacts an estimated 15% of couples worldwide [4]. RIF is described as the inability to conceive following the transfer of quality embryos into the endometrium through a minimum of three IVF procedures [1]. The causes of implantation failure have been identified as embryo factors, mother age, uterine factors, and multifactorial effectors [5, 6]. A dysfunctional immune response has been linked to recurrent miscarriages and peri‐implantation embryo failure in IVF patients [7]. Therefore, immunotherapy has been empirically used in couples with RIF based on data suggesting that the loss of embryo implantation may be caused by immunological issues [8]. Treatment options vary depending on what caused it and may include immunosuppressive medications, IVIg, low‐molecular‐weight heparin therapy, aspirin, and HCQ. To have a successful reproductive result, Th1 and Th2 cytokines must be balanced correctly [9, 10, 11].

In this case, our RIF patient was treated with HCQ, and her IVF result after treatment was successful. HCQ has therapeutic properties that include anti‐thrombotic, vascular protection, and immunomodulatory effects that may impact some immunological causes of RIF. It is among the medications proposed as an immunomodulator in the past few years to lessen immunologic basis infertility and conditions [12]. Many mechanisms have been proposed to explain how HCQ modulates immunity. Overall, the anti‐inflammatory and immunosuppressive characteristics of HCQ mediate its immunomodulatory actions, which can lead to a shift toward T‐reg responses that are beneficial for a successful implantation [8].

On the other side, there are several potentially harmful consequences of HCQ, including cutaneous responses, neuromuscular side effects, cardiac toxicities, QT prolongation, gastrointestinal disruption, and ocular problems [3]. The dermatologic reactions caused by HCQ can be classified into two groups based on the average cumulative dosage. When the mean cumulative dosage exceeds 100 g, the following adverse events are observed: dermatitis, stomatitis, cutaneous hyperpigmentation, pruritis, drug eruption, melanonychia, photosensitivity, and SJS/TEN. On the other hand, the following reactions, including AGEP, urticaria, psoriasis, DRESS, erythroderma, blistering, erythema multiforme, porphyria, and hair discoloration, occurred after a mean cumulative intake of less than 100 g [13]. It is important to note that HCQ is generally considered safe during pregnancy, and its use during lactation is also regarded as safe, with no significant adverse effects observed in the infant in most cases [2].

An uncommon adverse effect of HCQ is AGEP; even fewer cases have histologic evidence of AGEP and appear clinically as SJS or TEN. More classical AGEP linked to HCQ usually goes away fast if the drug is stopped [14]. Typically, most TEN cases present within the first 2 months of initiating the medication [15]. However, Callaly et al. reported a patient for more than 2 years using HCQ and now presented TEN [16]. Considering that more than 30% of the patient's total body surface area is involved, along with epidermal detachment, TEN is differentiated from SJS and SJS/TEN [17]. These variations highlight the importance of taking drug reactions into account when dealing with sudden skin conditions, especially in individuals taking HCQ.

We present a rare case of RIF, who experienced TEN as a side effect of HCQ. In our patient, who had been using HCQ for approximately seven weeks, the skin condition improved significantly within 3 days of discontinuing the medication. This strongly suggests that the diagnosis is attributable to a drug reaction caused by HCQ, although it is plausible that there may be other factors contributing to the development of TEN in this patient. Although no other specific encounter was identified in the patient's history, we acknowledge recall bias as a key limitation. Importantly, this is the first documented case of HCQ‐induced TEN in a patient with RIF, highlighting two critical imperatives: the ongoing vigilance required to monitor rare, delayed adverse events to prevent irreversible damage and the implementation of personalized medicine and education to optimize treatment efficacy and safety [18].

## Author Contributions

**Hatav Ghasemi Tehrani:** conceptualization, resources, supervision, validation. **Faezeh Zakerinasab:** writing – original draft, writing – review and editing. **Negar Khalili Geshnigani:** writing – original draft, writing – review and editing. **Rahem Rahmati:** data curation, validation, project administration, writing – review and editing. **Nastaran Zamani Dehkordi:** conceptualization, data curation, investigation, methodology, resources, writing – review and editing.

## Ethics Statement

This study was conducted in accordance with the Helsinki Declaration.

## Consent

Written informed consent was obtained from the patient for publication of this case report and any accompanying images.

## Conflicts of Interest

The authors declare no conflicts of interest.

## Data Availability

The data that support the findings of this study are available from the corresponding author upon reasonable request.
